# Ophthalmic ropinirole is an equally effective emetic agent in healthy dogs compared to intravenous apomorphine

**DOI:** 10.3389/fvets.2025.1554107

**Published:** 2025-03-17

**Authors:** Jack A. Lee, Katie Como, Xiaojuan Zhu, Julie Schildt

**Affiliations:** ^1^Department of Veterinary Clinical Sciences, School of Veterinary Medicine, Louisiana State University, Baton Rouge, LA, United States; ^2^Department of Small Animal Clinical Sciences, School of Veterinary Medicine and Biomedical Sciences, Texas A&M University, College Station, TX, United States; ^3^Office of Information Technology, University of Tennessee, Knoxville, TN, United States; ^4^Small Animal Clinical Sciences, College of Veterinary Medicine, University of Tennessee, Knoxville, TN, United States

**Keywords:** emesis, apomorphine, ropinirole, maropitant, metoclopramide, dog

## Abstract

**Introduction:**

Emesis is commonly induced in the veterinary setting due to toxin or foreign material ingestion. The dopamine agonist apomorphine is commonly used for this indication. The novel dopamine-2 specific agonist ropinirole was approved by the U.S. Food and Drug Administration for this indication in 2020. Data to compare the efficacy and adverse effects profile of these medications is important for clinical decision making.

**Methods:**

This blinded randomized crossover trial compared the efficacy of intravenous apomorphine to ophthalmic ropinirole in 24 healthy dogs. Factors assessed include efficacy at inducing vomiting within 20 min, need for redosing of the emetic agent, time to start of emesis, and incidence of adverse effects.

**Results:**

Both apomorphine and ropinirole were highly effective at emesis induction, with 95.8 and 100% success rates, respectively, and no difference between groups. Repeated dosing was needed after 20 min in 25% of the apomorphine group and 8.3% of the ropinirole group for successful emesis induction. Median time to onset was significantly shorter with apomorphine (1.18 min) than ropinirole (8.85 min). Incidence of adverse effects was similar, with a higher incidence of ocular redness and protracted vomiting in the ropinirole group.

**Conclusion:**

These results suggest similar efficacy of ropinirole compared to apomorphine, with similar adverse effect rates.

## Introduction

The induction of emesis is an invaluable tool in the management of canine patients who have ingested toxic substances or foreign materials (1, 2). Known or suspected ingestion of toxins remains the most common reason for the induction of emesis in dogs, though medically induced emesis can be also be useful in avoiding gastrointestinal obstructions secondary to foreign body ingestion (1, 3). Emesis induction is contraindicated in patients who have ingested caustic or corrosive substances, are showing signs associated with toxin exposure, have altered mentation, are predisposed to aspiration, are in respiratory distress, or have severe acid-base or electrolyte derangements that may be worsened by vomiting (4). Possible complications secondary to emesis induction include aspiration, lodging of foreign material in the esophagus/oropharynx, vasovagal events, esophagitis and associated stricture, intussusception, and plication of intestines in patients with linear foreign bodies (5).

The vomiting mechanism is initiated by the emetic center, which is composed of a group of nuclei found in the medulla oblongata of the brainstem (6). The most direct and rapid way to initiate the vomiting response is to stimulate the chemoreceptor trigger zone (CRTZ), which is located outside the blood-brain barrier and activates the emetic center through the humoral pathway (7). In the dog, the CRTZ is rich in dopaminergic (D_2_), histaminergic (H_1_), adrenergic (α_2_), serotonergic (5HT_3_), cholinergic (M_1_), enkephalinergic (ENK_μ, δ_), and neurokinergic (NK_1_) receptors (6). D_2_ receptor agonists are particularly potent emetics in dogs (2).

The optimal emetic agent is characterized by high efficacy, a rapid onset and short duration of action, ease of administration, and limited associated side effects. Apomorphine is a rapid-acting, synthetic opiate that is commonly used for emesis induction in dogs (4, 8). Apomorphine is a non-selective dopamine receptor agonist that triggers vomiting by stimulating D_2_ receptors in the CRTZ. Previous studies have demonstrated efficacy of between 80 and 97% (1, 3, 8–11). In addition to D_2_ receptors, apomorphine may activate other receptor classes, including 5HT and μ opioid receptors, particularly at higher doses or with repeated administration. Associated adverse effects may include sedation, respiratory depression, hypotension, and tachycardia. If severe, naloxone can be used to reverse opioid induced sedation and respiratory depression, but does not inhibit the emetic effects of apomorphine (12). Injectable and transmucosal administration have been described (3, 10). While apomorphine is a commonly used emetic agent in dogs, it is not licensed for use in the United States.

In 2020, an ophthalmic solution of ropinirole became the first U.S. Food and Drug Administration (FDA) approved emetic agent licensed for use in dogs (13). Ropinirole is a dopamine agonist with high selectivity for D_2_ receptors (2, 14). A randomized, double-blind controlled study in healthy dogs found that 95% of dogs treated with ropinirole vomited within 30 min of administration (2). As with apomorphine, the D_2_ receptor-associated side effects of ropinirole may include tachycardia and hypotension, but due to its increased selectivity, additional side effects may be limited and can be reversed with a dopamine receptor antagonist, such as metoclopramide. Other reported side effects are related to the route of administration, and include temporary conjunctival hyperemia, third eyelid protrusion, ocular discharge, and blepharospasm (14). Recently, Rosenstein et al. (11) performed a non-randomized non-controlled trial of the efficacy of ropinirole in clinical patients, which noted 91.4% efficacy and a 17% incidence of adverse effects, including protracted vomiting, ocular irritation, and sedation, which were self-limiting.

As a novel agent, literature on the use of ropinirole in dogs is limited. The purpose of this study was to compare the efficacy of emesis induction between intravenous apomorphine and ophthalmic ropinirole and to describe any adverse effects associated with these agents when administered to healthy dogs. Based on previously published data, we hypothesized that there would be no difference in emetic rate between ropinirole and apomorphine.

## Methods

Sample size calculations were performed to demonstrate non-inferiority (within 10% efficacy) of ropinirole compared to apomorphine based on 95% efficacy of apomorphine, with 80% power. This demonstrated the need for 30 dogs. Due to constraints on available dogs, a sample size of 24 dogs was used, which has >70% power to detect this difference and was still considered to provide clinically meaningful results.

The animal study was reviewed and approved by the University of Tennessee Institutional Animal Care and Use Committee (protocol # 2902-0522). For client owned animals, written informed consent was obtained from the owners for the participation of their animals in this study.

Twenty-two purpose bred healthy dogs from the university teaching and research colony were enrolled. During the time of the study, they were housed individually. Two healthy-client owned dogs were also enrolled to meet necessary sample sizes. These dogs were housed with their owners until the 2 days of data collection.

This was a blinded randomized crossover design, with each dog receiving both study drugs, with 7 days of washout between study days.

A physical examination was performed, and weight, temperature, heart rate, and respiratory rate were measured on each dog the morning of data collection. Half of the normal amount of food was given to all dogs at least 3 h prior to the start of the study protocol. Dogs were randomized to receive either apomorphine hydrochloride (Letco Medical, Decatur, AL, USA) 0.03 mg/kg IV in a cephalic vein or ropinirole (Vetoquinol USA, Fort Worth, TX, USA) 3.75 mg/m^2^ ophthalmically dosed according to manufacturer dosing chart (15). The investigators remained blinded to which drug the dogs had received. Dogs in the apomorphine group had a small compression bandage placed over the injection site. To facilitate continued blinding of the investigators, dogs in the ropinirole group also had a compressive bandage placed over the cephalic vein of one forelimb. To maintain blinding, dogs in the apomorphine group also received saline eye drops (the number of which was equivalent to the dog's ropinirole dose).

If no emesis had occurred within 20 min, the study drug was re-dosed a single time. This was based on the ropinirole manufacturer's instructions (15). If >3 episodes of emesis occurred, an anti-emetic was administered. Dogs that had received apomorphine received maropitant 1 mg/kg SC. Dogs that had received ropinirole received metoclopramide 0.5 mg/kg SC, per the ropinirole manufacturer recommendations. Investigators remained blinded as to which anti-emetic was being administered. All dogs received an anti-emetic after 60 min if they had not already.

Each dog was continually monitored for 1 h after drug administration, with heart rate and any side effects noted at 15, 30, 45, and 60 min after study drug administration. Time of emesis, number of emetic events, time between first and last episode of emesis (duration of emesis), whether drug redosing was required, and whether an anti-emetic was administered was recorded. Side effects monitored for included persistent nausea, lip licking, hypersalivation, retching, sedation, hyperexcitability, eye redness, squinting, ocular discharge, peri-ocular swelling, and “other”. Naloxone was available for administration in case of excess sedation, hyperexcitability, or bradycardia (< 60 beats per min) lasting for longer than 15 min after receiving apomorphine.

Client owned dogs were returned to their owners 6 h after emesis. A snack (1/3–1/2 of normal meal size) was offered to all dogs 6 h after emesis. A follow up physical exam was performed on all dogs 24 h after emesis.

### Statistical analysis

For categorical data, a 2-tailed Fisher's exact test was performed when group size was ≤ 5. A Pearson's chi-squared test was used when *n* > 5 for each group. Summary statistics with counts, median, and quartiles were also generated. For numerical data, a Shapiro-Wilk test was used to assess for normality. A Wilcoxon paired ranks test was used for non-parametric data and a paired *t*-test used for parametric data. A two-way mixed effect ANOVA was performed to determine the effect of each drug on heart rate over time. Least square means were computed and separated with the Tukey's HSD correction method. All statistical analysis was performed with JMP Pro 16.0 (SAS Institute Inc., Cary, NC). *P* < 0.05 was considered as significant.

## Results

### Demographics

Twenty-four dogs were enrolled. Twelve female spayed and 12 male neutered. Twenty were Beagles, two were hound mixes, one was a Chihuahua mix and one an Australian Cattle Dog mix. Median age 3.2 years (range 2.4–8 years). Median weight 12.5 kg (range 10–23.2 kg).

### Emesis induction

There was no difference in overall efficacy between apomorphine and ropinirole (95.8 vs. 100%, respectively) (Table 1). Only one dog (in the apomorphine group) failed to vomit after up to two doses. There was not a significant difference between groups in need for emetic redosing (25 vs. 8.3%). Dogs in the apomorphine group vomited more rapidly, and had a shorter duration of vomiting (Table 2). Dogs in the ropinirole group vomited more often than in the apomorphine group (median 4 times vs. 2.5 times, respectively). Early anti-emetic administration due to >3 episodes of vomiting was more frequent in the ropinirole group (Table 1). In dogs receiving an early anti-emetic, all vomiting ceased within 3.17 min.

**Table 1:** Emesis success, need for repeated emetic dosing, and need for early anti-emetic administration in 24 healthy dogs given IV apomorphine and ophthalmic ropinirole.

	**Apomorphine**	**Ropinirole**	
**Variable**	***N*** **(%)**	***N*** **(%)**	***p*** **-value**
Emesis success	23 (95.8)	24 (100)	1
Need for repeat emetic dosing	6 (25)	2 (8.3)	0.24
Anti-emetic given early (>3 episodes of emesis)	7 (29.2)	21 (87.5)	**< 0.0001**

Values where *p* < 0.05 are bolded.

**Table 2:** Number of episodes of emesis, time to first emesis, time to first emesis in dogs requiring drug redosing, and duration of vomiting in 24 healthy dogs given IV apomorphine and ophthalmic ropinirole.

	**Apomorphine**		**Ropinirole**		
**Variable**	***N***	**Median**	***N***	**Median**	***p*** **-value**
Episodes of emesis	24	2.5	24	4	**< 0.0001**
Time to first emesis (min)	23	01:30	24	7:37	**0.0004**
Time to first emesis if redosed (min)	5	1:11	2	8:51	N/A
Duration of vomiting (min)	17	1:13	24	4:53	**< 0.0001**

Values where *p* < 0.05 are bolded.

### Adverse effects

A two-way mixed effect measures ANOVA revealed that there was not a statistically significant interaction between the effects of emetic agent and time measurement (*p* = 0.36). There was a statistically significant time effect on heart rate (*p* = 0.002). Heart rate was significantly lower at 45 min than baseline, with no effect of drug on heart rate (*p* = 0.89; Table 3).

**Table 3:** ANOVA effect of time on heart rate in 24 healthy dogs given IV apomorphine and ophthalmic ropinirole.

**Category**	**Mean (bpm)**	**Std. deviation**	**Letter groups**
HR at baseline	116.3	22.5	A
HR at 15 min	117.9	26	A
HR at 30 min	107.4	22	AB
HR at 45 min	104.5	17.4	B
HR at 60 min	109.7	24.1	AB
HR at 24 h	108.9	20.1	AB

HR, heart rate; BPM, beats per minute.

If two categories share the same letter they are not statistically different from another one. Otherwise, two categories without the same letters are significantly different from one another.

Dogs in the ropinirole group had a higher incidence of eye redness (either conjunctival or scleral erythema) (Table 4). Two dogs had eye redness and two had ocular discharge before study drug administration, so were excluded from this portion of analysis. There was no difference in frequency of nausea, lip licking, hypersalivation, retching, sedation, hyperexcitability, squinting, ocular discharge, or peri-ocular swelling. Single other events in the apomorphine group included a hunched abdomen after vomiting, excessive swallowing, and a transient increased respiratory effort after injection. Other events in the ropinirole group included scratching at face, excessive panting, increased thirst, excessive blinking after redosing of the medication, and two episodes of head shaking.

**Table 4:** Adverse effects seen in 24 healthy dogs given IV apomorphine and ophthalmic ropinirole.

	**Apomorphine**	**Ropinirole**	
**Variable**	***N*** **(%)**	***N*** **(%)**	***p*** **-value**
Nausea	17 (70.8)	18 (75)	0.75
Lip licking	20 (83.3)	22 (91.7)	0.66
Hypersalivation	6 (25)	7 (29.2)	0.75
Retching non-productively	13 (54.2)	18 (75)	0.13
Sedation	7 (29.2)	6 (25)	0.75
Hyperexcitability	0 (0)	0 (0)	N/A
Eye redness	2 (8.3)	9 (37.5)	**0.036**
Squinting	0	2 (0.49)	0.49
Ocular discharge	3 (12.5)	4 (16.7)	1
Peri-ocular swelling	1 (4.2)	1 (4.2)	1

Values where *p* < 0.05 are bolded.

In addition, three dogs that received ropinirole vomited between 4 and 12 h after drug administration. Two of these dogs were noted to seem persistently nauseous during the afternoon after ropinirole administration. The third vomited overnight and seemed normal when assessed in the morning. All three dogs received 1 mg/kg doses of maropitant subcutaneously and had returned to normal with normal appetites by 24 h after study drug administration.

No dogs had prolonged anorexia beyond 24 h or required any additional supportive care. Physical exam 24 h after emesis induction did not reveal any significant persistent abnormalities. No dogs required naloxone.

## Discussion

In this blinded randomized crossover trial in healthy dogs, ophthalmic ropinirole was an equally effective emetic agent compared to IV apomorphine. The ideal emetic agent would be highly effective, with a low need for repeated doses. Both were highly effective, 100 and 95.8%, respectively. 70.8% of dogs vomited within 20 min of receiving apomorphine, while 91.7% vomited within 20 min of receiving ropinirole. The high efficacy of apomorphine in this study was comparable to previous studies, which has been reported between 80 and 97% effective (3, 9, 10). The efficacy of ropinirole in this population is also similar to the 91.4–95% rate previously reported (2, 11).

Both agents induced emesis rapidly, each with a median time under 8 min, which is likely to be clinically acceptable in most situations. The median time to emesis was ~6 min shorter with apomorphine, which could be clinically relevant in certain indications, such as rapidly absorbed toxins. Similarly, Manley et al. (16) found more dogs vomited within 10 min of receiving IV apomorphine than ophthalmic ropinirole. Time to emesis with apomorphine depends on route of administration, with time of onset of 2 and 13.5 min reported for IV and SC administration, respectively (3, 17). Previous work reported a time of 11–12 min (range 2–75 min) with ropinirole (2, 11). Most dogs in the present study required only a single dose of the emetic. Those that had to receive a second dose usually vomited within about 5 min.

In this study, the authors chose to administer anti-emetic agents after four vomiting episodes to avoid prolonged discomfort for the animals involved, as based on the authors' experience it was considered unlikely that further episodes would result in clinically significant recovery of ingesta or provide additional data of interest. This precludes full assessment of the number of vomiting episodes caused by each medication. However, dogs receiving ropinirole were more likely to exceed four emetic episodes and require early administration of an anti-emetic. Previous work showed a median of 4 vomiting episodes (range 1–13), with a mean duration of 23 min (range 0–108 min) (2). The emetic action of apomorphine is generally self-limiting due to eventual binding of apomorphine to opioid receptors within the brain, which has an anti-emetic effect. A previous study of IV apomorphine found a median of 3 episodes of vomiting with a maximum of 6 episodes (3). In another study, mean duration of vomiting after apomorphine administration was 27 min (18).

During the 1-h period of direct observation, the only side effect that differed between groups was the incidence of eye redness with ropinirole. This effect was self-resolving by the following day in this population. It has been hypothesized that this effect may be due to direct irritation from the drug or its carrier, but may also be related to ocular dopamine receptor activation (19). Dopamine receptor antagonists may speed resolution (2). Common previously reported ophthalmic effects of ropinirole include conjunctival hyperemia (51%), protrusion of the third eyelid (38%), conjunctival discharge (30%), and blepharospasm (19%). This has been consistent across studies (11). The use of ropinirole ophthalmic drops is contraindicated in cases of corneal ulceration, ocular irritation, or ocular injury (2). No other ophthalmic-related side effects were more common in the ropinirole group. Patients in the apomorphine group did receive saline eyedrops to keep the investigators blinded, which may have increased the incidence of ocular discharge. Dogs with ocular discharge or reddening prior to medication administration were not included in analysis of those groups, which could have affected the frequencies reported.

Sedation was a common systemic side effect of both medications, consistent with previous reports. This is likely due to dopamine stimulation by both drugs, as well as the cross reactivity of apomorphine with other receptors, including μ opioid and 5HT-3 receptors (8). Tachycardia is also a commonly reported effect of both medications, again due to dopamine stimulation. In this population, the majority of animals were tachycardic prior to medication administration, which limits assessment of drug induced tachycardia. This high heart rate at baseline is likely due to excitement or anxiety, given that the presence of the study personnel was a deviation in the normal routines of the colony dogs, and caused visible excitation and vocalization. For the client-owned dogs, being in the hospital was also a change in routine. An acclimatization period was not used prior to heart rate collection, which could have helped to clarify this issue. Heart rate was slightly lower at 45 min, but again returned to baseline at 60 min. Measuring heart rate every 15 min via auscultation during the monitoring period also caused visible excitement, which could have falsely increased heart rates, though the effects of the medications could have played a role. It has been hypothesized that the greater D2 selectivity of ropinirole will decrease side effects of administration. In this population, such a difference was not seen, though all effects were mild and self-limiting.

Three dogs in the ropinirole group had additional episodes of vomiting between 4 and 12 h after drug administration, despite receiving metoclopramide. Previous literature has not consistently reported protracted vomiting. In the study by Suokko et al. (2), no dogs vomited beyond 108 min after ropinirole administration, and no animals that received metoclopramide had additional vomiting episodes. However, Rosenstein et al. (11) reported protracted vomiting in a small number of dogs. The manufacturer recommends metoclopramide at 0.5 mg/kg IV or SC to treat protracted vomiting, as well as to decrease the prevalence of most ropinirole-associated clinical signs (15). Metoclopramide is a D_2_ receptor antagonist, acting on the CRTZ. It also weakly antagonizes serotonin 5-HT_3_ receptors and agonizes 5-HT_4_ receptors, which can cause sedation, antiemetic, and extrapyramidal signs. These serotonin effects provide additional peripheral anti-emetic effects. It has additional promotility effects on the upper GI tract through increased sensitization of smooth muscle cells to acetylcholine and 5-HT_4_ (20–23). Half-life in dogs after oral administration is 1–2 h (14). The pharmacokinetics of subcutaneous metoclopramide is not well understood, and appears to have intra-individual variation. Clinical studies have demonstrated its efficacy (20). It was shown to reduce emesis from morphine even while given simultaneously, though it was significantly more effective when given 30 min prior (23). Ropinirole has a half-life of ~4 h with IV administration. Metabolism is via hepatic biotransformation with urinary excretion. No direct correlation between the drug concentration in plasma and the duration of vomiting was observed after ocular administration (15). It is therefore possible that that effective duration of subcutaneous metoclopramide was shorter than that of ropinirole. It is also possible that the nausea from earlier emetic episodes persisted. Alternatively, other causes of vomiting such as stress from study manipulation, changes in normal feeding schedule, or other underlying gastrointestinal disorders could have served as a trigger. No dogs otherwise vomited during the week surrounding the study.

Maropitant, a neurokinin-1 antagonist, was used as the anti-emetic after apomorphine administration given literature to support its efficacy and its common use in clinical practice (20, 22, 24). Neurokinin-1 antagonism in both the emetic center and GI tract inhibits substance P to prevent vomiting. Findings on the use of maropitant with ropinirole administration have not been published. Half-life after SC administration is 7.75 h, with maximum plasma levels within 45 min. Elimination is via the hepatic cytochrome P450 system (8, 25). It has been shown to reduce vomiting due to morphine by 70% when given subcutaneously 30 min before (20). However, it had no effect when given simultaneously (26). Other studies also showed efficacy when given 45 and 60 min prior to exposure (20, 27). It is therefore most likely that further episodes of emesis were prevented by the self-limiting nature of apomorphine rather than immediate effect of the drug, though administration can prevent ongoing emesis. It is unknown whether administration of maropitant would have prevented ongoing vomiting in the ropinirole group, though this could be considered given the more prolonged duration of action.

There are several limitations to this study. Power analysis suggested that the available sample size would give 70% power to detect a difference in the primary outcome (emesis success) between the two drugs. A type II error is possible. The study may have also been underpowered to detect a difference in adverse effects profile or detect rare effects. Additionally, this study was performed in healthy animals, primarily from a research and teaching colony. Efficacy and side effects may differ in a clinical population.

There remains a paucity of information on use of ropinirole in clinical patients, as well as those with comorbidities, which should be addressed to help guide clinical decision making. Further information on the use of anti-emetics after ropinirole administration would also be valuable given the protracted vomiting noted here. In patients with toxin ingestion, protracted vomiting would be a concern if oral therapies such as activated charcoal are administered. As the only FDA approved emetic agent in dogs, veterinarians, particularly in the United States, may face restrictions on the use of other agents, which should be considered and drive continued research.

In conclusion, both IV apomorphine and ophthalmic ropinirole were highly effective at inducing vomiting in healthy dogs. Onset of vomiting was more rapid with apomorphine, while ropinirole caused more episodes of vomiting. Side effects profiles were similar other than increased ocular redness and several episodes of protracted vomiting in dogs receiving ropinirole. Further study is warranted.

## Data Availability

The original contributions presented in the study are included in the article/supplementary material, further inquiries can be directed to the corresponding author.

## References

[1] Yam E Hosgood G Smart L. Comparison of the use of sodium carbonate (washing soda crystals) and apomorphine for inducing emesis in dogs. Aust Vet J. (2016) 94:474–7. 10.1111/avj.12530 27891595

[2] Suokko M Saloranta L Lamminen T Laine T Elliott J. Ropinirole eye drops induce vomiting effectively in dogs: a randomised, double-blind, placebo-controlled clinical study. Vet Rec. (2020) 186:283. 10.1136/vr.104953 31409749 PMC7063390

[3] Fischer C Drobatz KJ Thawley VJ. Evaluation of subcutaneous vs. intravenous administration of apomorphine for induction of emesis in dogs. J Am Vet Med Assoc. (2021) 259:283–7. 10.2460/javma.259.3.283 34242075

[4] Humm K Greensmith T. Intoxication in dogs and cats: a basic approach to decontamination. In Pract. (2019) 41:301–8. 10.1136/inp.l5062

[5] Zersen KM Peterson N Bergman PJ. Retrospective evaluation of the induction of emesis with apomorphine as treatment for gastric foreign bodies in dogs (2010-2014): 61 cases. J Vet Emerg Crit Care. (2020) 30:209–12. 10.1111/vec.12942 32077200

[6] Gallagher A. Vomiting and regurgitation. In:Ettinger S Feldman EC Cote E, editors. Textbook of Veterinary Internal Medicine: Diseases of the Dog and the Cat. 18 ed. St. Louis, Mo: Elsevier Saunders (2017). p. 160–3.

[7] Elwood C Devauchelle P Elliott J Freiche V German AJ Gualtieri M. Emesis in dogs: a review. J Small Anim Pract. (2010) 51:4–22. 10.1111/j.1748-5827.2009.00820.x 20137004 PMC7167204

[8] Plumb D. Apomorphine. Plumb's Veterinary Drugs.Updated February (2022). Available online at: https://app.plumbs.com/drug-monograph/9113HOC31WPROD?source=search&searchQuery=apomorphine (accessed August 3, 2022).

[9] Humphrey SJ Turman CN Curry JT Wheeler GJ. Cardiovascular and electrocardiographic effects of the dopamine receptor agonists ropinirole, apomorphine, and PNU-142774E in conscious beagle dogs. J Cardiovasc Pharmacol. (2006) 47:337–47. 10.1097/01.fjc.0000205983.05771.f5 16633074

[10] Eurell TE Peacock RE. Induction of emesis with apomorphine using a novel gingival administration method in dogs. J Vet Emerg Crit Care. (2021) 31:795–9. 10.1111/vec.13115 34433235

[11] Rosenstein NA Johnson JA Kirchofer KS. Ropinirole has similar efficacy to apomorphine for induction of emesis and removal of foreign and toxic gastric material in dogs. J Am Vet Med Assoc. (2023) 261:1140–6. 10.2460/javma.23.01.0027 37072118

[12] Rosendale ME. Decontamination strategies. Vet Clin North Am Small Anim Pract. (2002) 32:311–21. 10.1016/S0195-5616(01)00007-9 12012737

[13] U.S. Food & Drug Administration. FDA Approves Clevor (Ropinirole Ophthalmic Solution) to Induce Vomiting in Dogs (2020). Available online at: https://animaldrugsatfda.fda.gov/adafda/app/search/public/document/downloadFoi/9248 (accessed December 12, 2024).

[14] Plumb D. Ropinirole Plumb's Veterinary Drugs (2022). Available online at: https://app.plumbs.com/drug/1VSUrGkcww7JIw121bjLqA?source=search&searchQuery=ropinie (accessed August 3, 2022).

[15] Vetoquinol. Clevor. Fort Worth, TX: Vetoquinol.

[16] Manley SR Berg AN Rozanski EA Sweigart BA Lynch AM. Intranasal and intravenous apomorphine outperform ropinirole ocular drops for induction of emesis in dogs within ten minutes: a randomized, controlled clinical trial. J Am Vet Med Assoc. (2024) 262:635–9. 10.2460/javma.23.11.0628 38452486

[17] Scherkl R Hashem A Frey HH. Apomorphine-induced emesis in the dog—routes of administration, efficacy and synergism by naloxone. J Vet Pharmacol Ther. (1990) 13:154–8. 10.1111/j.1365-2885.1990.tb00763.x 2384906

[18] Khan SA McLean MK Slater M Hansen S Zawistowski S. Effectiveness and adverse effects of the use of apomorphine and 3% hydrogen peroxide solution to induce emesis in dogs. J Am Vet Med Assoc. (2012) 241:1179–84. 10.2460/javma.241.9.1179 23078563

[19] Kerry PJ Wakefield ID Evans JG. Ocular changes induced in the beagle dog by intravenous infusion of a novel dopaminergic compound, FPL 65447. Toxicol Pathol. (1993) 21:274–82. 10.1177/019262339302100302 7902612

[20] Lorenzutti AM Martín-Flores M Litterio NJ Himelfarb MA Invaldi SH Zarazaga MP. Comparison between maropitant and metoclopramide for the prevention of morphine-induced nausea and vomiting in dogs. Can Vet J. (2017) 58:35–8. Available online at: https://pmc.ncbi.nlm.nih.gov/articles/PMC5157735/#:~:text=The%20efficacy%20of%20MCP%20as,in%20accord%20with%20previous%20findings 28042152 PMC5157735

[21] Plumb D. Metoclopramide. Plumb's Veterinary Drugs (2022). Available online at: https://app.plumbs.com/drug-monograph/9113HOC31WPROD?source=search&searchQuery=apomorphine (accessed October 31, 2022).

[22] Sedlacek HS Ramsey DS Boucher JF Eagleson JS Conder GA Clemence RG. Comparative efficacy of maropitant and selected drugs in preventing emesis induced by centrally or peripherally acting emetogens in dogs. J Vet Pharmacol Ther. (2008) 31:533–7. 10.1111/j.1365-2885.2008.00991.x 19000276

[23] Brioschi FA Gioeni D Jacchetti A Carotenuto AM. Effect of metoclopramide on nausea and emesis in dogs premedicated with morphine and dexmedetomidine. Vet Anaesth Analg. (2018) 45:190–4. 10.1016/j.vaa.2017.09.042 29409803

[24] Claude AK Dedeaux A Chiavaccini L Hinz S. Effects of maropitant citrate or acepromazine on the incidence of adverse events associated with hydromorphone premedication in dogs. J Vet Intern Med. (2014) 28:1414–7. 10.1111/jvim.12414 25146756 PMC4895585

[25] Benchaoui HA Cox SR Schneider RP Boucher JF Clemence RG. The pharmacokinetics of maropitant, a novel neurokinin type-1 receptor antagonist, in dogs. J Vet Pharmacol Ther. (2007) 30:336–44. 10.1111/j.1365-2885.2007.00877.x 17610407

[26] Lorenzutti AM Martín-Flores M Litterio NJ Himelfarb MA Zarazaga MP. Evaluation of the antiemetic efficacy of maropitant in dogs medicated with morphine and acepromazine. Vet Anaesth Analg. (2016) 43:195–8. 10.1111/vaa.12286 26095960

[27] Hay Kraus BL. Efficacy of maropitant in preventing vomiting in dogs premedicated with hydromorphone. Vet Anaesth Analg. (2013) 40:28–34. 10.1111/j.1467-2995.2012.00788.x 23082784

